# Impact of spontaneous recanalization of occlusive cervical artery dissection on risk of stroke

**DOI:** 10.1007/s00415-025-13371-y

**Published:** 2025-09-11

**Authors:** Lukas Mayer-Suess, Josefin E. Kaufmann, Lukas Scherer, Anel Karisik, Malik Galijasevic, Stephanie Mangesius, Elke Ruth Gizewski, Stefan Kiechl, Christopher Traenka, Stefan T. Engelter, Michael Knoflach

**Affiliations:** 1 Department of Neurology, Medical University Innsbruck, Innsbruck, Austria; 2 Department of Neurology, University Hospital Basel and University of Basel, Basel, Switzerland; 3 VASCage, Research Center On Vascular Ageing and Stroke, Innsbruck, Austria; 4 Department of Radiology, Medical University Innsbruck, Innsbruck, Austria; 5 Neuroimaging Research Core Facility, Medical University of Innsbruck, Innsbruck, Austria

**Keywords:** Cervical artery dissection, Recanalization, Ischemic stroke

## Abstract

**Introduction:**

Occlusive cervical artery dissection (CeAD) is associated with worse patient outcome. The net clinical benefit of acute revascularization measures has to be weighed against the likelihood of spontaneous recanalization. Our aim was to assess the hitherto un-addressed impact of spontaneous recanalization on stroke risk in patients with occlusive CeAD.

**Methods:**

MRI verified CeAD patients with initially occlusive CeAD within cohort study that did not undergo acute revascularization measures were assessed. Follow-up data derived from clinical routine and study specific assessments. Outcomes of interest were occurrence of (i) recanalization and (ii) ischemic stroke upstream of CeAD-related occlusion. Adjusted logistic regression analysis addressed the impact of recanalization on said outcomes.

**Results:**

97/328 (29.6%) patients had occlusive CeAD and did not undergo acute revascularization treatment. Upon follow-up, 56/97 (57.7%) showed spontaneous recanalization of initially occlusive CeAD. Female sex (OR 0.41[0.18, 0.97]; *P* = 0.043) and internal carotid artery dissection (OR 0.33[0.14, 0.78]; *P* = 0.012) were the only factors independently associated with recanalization. Within a median follow-up of 8.2 (1.58, 12.8) years, a total of 18/97 (18.6%) patients suffered ischemic stroke upstream of the initially CeAD-affected vessel. After adjusting for confounders, spontaneous recanalization was independently associated with lower rates of cerebral ischemia upon follow-up (OR 0.28[0.09, 0.90]; *P* = 0.032), most notably also independent of type of antithrombotic treatment.

**Conclusions:**

Spontaneous recanalization in occlusive CeAD is associated with lower rates of stroke upon follow-up. These results indicate that persistent CeAD-related occlusion remains a risk-factor for recurrent ischemic events, thus calling for future trials addressing optimal medical treatment.

**Trial registration:**

N/A.

**Guarantor:**

Lukas Mayer-Suess.

**Supplementary Information:**

The online version contains supplementary material available at 10.1007/s00415-025-13371-y.

## Introduction

Care for patients with cervical artery dissection (CeAD), one of the main reasons for ischemic stroke in young individuals (< 55 years old), remains challenging for stroke physicians [1, 2]. This is especially true if the dissection causes cervical vessel occlusion. In this case, eligibility for acute revascularization measures, such as endovascular thrombectomy (EVT), are still debated [3–5]. Emergent endovascular stenting seems to also only impact recanalization rate, while patient outcome is not improved [6]. Concerning medical treatment, optimal care (i.e. antiplatelets or anticoagulation) in CeAD has not yet been established [2, 7–9]. The fact that CeAD-related vessel occlusions show a considerable spontaneous recanalization rate can further complicate clinical decision making on optimal treatment [2, 10–13]. The uncertainty in optimal management in these patients is troublesome. Acute and residual occlusion due to CeAD has been associated with worse outcome, which relates to the poorer performance of acute revascularization measures in the acute phase and, upon long-term follow-up, is associated with a higher risk of recurrent ischemic stroke due to impaired collateral status or occurrence of stump embolization. [14, 15] As studies on invasive acute revascularization measures predominate, our goal was to shed light on whether spontaneous recanalization (i.e. in those not undergoing acute revascularization procedures like i.e. intravenous thrombolysis, EVT or stenting) of occlusive CeAD has an effect on occurrence of unfavorable clinical outcomes.

## Methods

### Patient recruitment and selection

The PROspective disSECTion study (ProSect) is a prospective, observational, single-center cohort study recruiting and following-up all CeAD patients treated at the Medical University of Innsbruck from 1996 onward. ProSect has, through prior retrospective studies, established a clinical care pathway for CeAD patients at the study site, including sequential MR-imaging-, neurovascular ultrasound- as well as clinical assessments and biosample collection. [16–18] All ProSect patients underwent said measures at baseline, 3- and 12-month follow-up and annually thereafter at a minimum. In addition, all electronically available medical data, including clinical and imaging assessments during in-hospital stay as well as during outpatient care performed outside of the ProSect specific care pathway, were recorded. To be eligible for the ProSect study, dissection had to be verified by visualization of vessel wall hematoma in T1 fat saturated MRI in the acute phase. For the current analysis, all ProSect patients were screened concerning their initial vessel pathology (i.e. occlusive or nonocclusive) and whether they underwent acute recanalization strategies (i.e. iv. thrombolysis/EVT/stenting or conservative treatment). In individuals with initial occlusion, all cerebrovascular imaging files, derived from both clinical routine as well as study specific time points, were screened by two independent raters to establish evidence of recanalization. Further, available electronic medical files and in-person follow-up visits were evaluated in regards of our outcome parameters.

### Variable definitions and statistical methodology

Patients with initial occlusive CeAD, which had to be confirmed in two independent imaging modalities (e.g. MRA and ultrasound or CTA and MRA) that did not undergo acute revascularization procedures and had a minimum of one follow-up were considered as our study cohort. Date of hospital admission due to CeAD was defined as baseline. Two types of follow-ups were considered: (1) *vessel recanalization follow-ups*: all cerebrovascular imaging assessments (MRA, CTA, neurosonography) were evaluated concerning the recanalization of initially CeAD-related occlusion. Recanalization was defined as restauration of antegrade blood-flow independent of residual stenosis, but excluding pseudo-occlusions, visualized by either MRA, CTA, DSA or ultrasound and adjudicated by two independent raters. (2) *Clinical outcome follow-up*: both clinical appraisal as well as MRI imaging had to be available. Herein, occurrence of novel cerebral ischemia with (symptomatic) or without (silent) clinical symptoms was recorded. Cerebral ischemia was defined as ischemic lesion or retinal infarction. For the sake of our study, only ischemia corresponding to the initial CeAD-related vessel occlusion were considered. All outcomes had to be adjudicated by a stroke physician as well as an experienced neuroradiologist.

Clinical presentation at baseline included evidence of cerebral ischemia (ischemic stroke or transient ischemic attack) as well as local symptoms (head/neck pain, Horner’s syndrome, tinnitus, cranial nerve palsy), which both had to be adjudicated by a stroke physician. TIA was defined as transient deficits due to cerebral ischemia without evidence of cerebral infarction (tissue-based definition). Clinical and functional status of patients were evaluated using NIHSS and mRS. Imaging characteristics included CeAD localization (i.e. internal carotid or vertebral artery), multiple vessel CeAD defined as more than one cervical artery showing mural hematoma in T1-fat saturated MRI sequences during admission, as well as multivessel segment (i.e. long-distance) occlusions. Minor trauma was defined as recent head/neck trauma without concomitant internal or external signs of injury. The recent infection was considered as respiratory infection ≤ 4 weeks prior to CeAD-related admission. Medical treatment was categorized in medication at admission (i.e. prior to CeAD admission) as well as hyperacute management (i.e. first antithrombotic treatment administered at CeAD-related hospital admission) and at hospital discharge.

For the current analysis, the study cohort was grouped to either (1) patients with recanalization of initially CeAD-related vessel occlusion or (2) without such recanalization during follow-up. Timing of recanalization was dichotomized as (1) early (i.e. during initial hospital stay) and (2) late (i.e. after discharge).

Variables were summarized as count (percentage) or median (1st quartile, 3rd quartile). Chi^2^, Mann–Whitney *U* and Kruskal–Wallis test were used in group comparisons, where applicable. Logistic regression analysis, applying adjustment for confounders (i.e. characteristics showing *P* < 0.05 in univariate comparison), was used to assess the impact of recanalization overall as well as recanalization time-point (i.e. early vs. late) on our individual outcome parameters. The results are presented as odds ratio (OR) and 95% confidence interval (CI). *P* values were two-sided and an alpha level of 0.05 is used. Analysis was conducted using IBM SPSS Statistics (IBM Corp. Released 2023. IBM SPSS Statistics for Windows, Version 29.0.2.0 Armonk, NY: IBM Corp).

### Standard protocol approvals, registration, and patient consents

All analyses were approved by the local ethics committee at the Medical University Innsbruck and appropriate informed consent of patients who took part in the ProSect-Study was obtained (EK# 1240/2018).

### Data availability

Anonymized data not published within this article will be made available to any qualified investigator upon reasonable request after ethics approval and receipt of a signed data transfer agreement. The current manuscript adheres to reporting guidelines of observational cohorts [19].

## Results

A total of 328 CeAD patients were included in the ProSect study with 116 (35.7%) having an initially occlusive vessel pathology. 3 of our 328 (0.9%) patients died during hospital stay, 2 of which did not have an initially occlusive CeAD-related vessel pathology. Differences between those with and without initial occlusion as well as our conservatively treated occlusive CeAD patients and those undergoing acute revascularization measures are given in the supplementary material (Tables S-1 and S-2). In short, those with initial CeAD-related occlusion were older, more likely to suffer cerebral ischemia, had higher NIHSS as well as mRS scores at admission, and were more likely to undergo acute revascularization treatments (thrombolysis and/or EVT). Stenting of CeAD-related pathologies was rare in both groups. Further, those undergoing acute revascularization measures less frequently reported local symptoms and more likely to have internal carotid artery CeAD. Of note, those undergoing acute revascularization presented with significantly more severe symptoms of ischemic stroke than those treated conservatively (Table S-2).

Of those 97 of 116 (83.6%) patients with initial occlusion managed conservatively, 56 (57.8%) showed spontaneous recanalization of CeAD-related occlusion on the long-run. Table 1 presents baseline characteristics of our entire cohort as well as those with and without recanalization.

**Table 1:** Patient and treatment characteristics of patients with initial occlusion as well as comparison between those with and without spontaneous recanalization

	Initial occlusion	No recanalization	Recanalization	*P* value
*N*	97	41 (42.3)	56 (57.7)	
***Patient characteristics***				
Age^†^	46.6 (39.8, 52.3)	47.7 (43.2, 52.0)	45.9 (35.9, 52.5)	0.165
Male^*^	57 (58.8)	29 (70.7)	28 (50.0)	0.032
mRS prior to CeAD^†^	0.0 (0.0, 0.0)	0.0 (0.0, 0.0)	0.0 (0.0, 0.0)	0.694
***Medication at admission***				
Antiplatelets^*^	2 (2.1)	2 (4.9)	0 (0.0)	0.186
Antihypertensives^*^	12 (12.4)	7 (17.1)	5 (8.9)	0.211
Statin^*^	4 (4.1)	3 (7.3)	1 (1.8)	0.217
***Clinical presentation***				
Ischemia^*^	84 (86.6)	39 (95.1)	45 (80.4)	0.047
NIHSS admission^†^	1.0 (0.0, 3.0)	1.0 (0.0, 3.0)	1.0 (0.0, 3.0)	0.697
mRS admission^†^	2.0 (1.0, 3.0)	2.0 (1.0, 3.0)	2.0 (1.0, 2.5)	0.321
Local symptoms^*^	78 (80.4)	32 (78.0)	46 (82.1)	0.448
Head/neck pain^*^	77 (79.4)	31 (75.6)	46 (82.1)	0.328
Horner’s^*^	14 (14.4)	4 (9.8)	10 (17.9)	0.212
Tinnitus^*^	1 (1.0)	0 (0.0)	1 (1.8)	0.581
Cranial nerve palsy^*^	4 (4.1)	2 (4.9)	2 (3.6)	0.560
Multiple vessel CeAD^*^	15 (15.5)	6 (14.6)	9 (16.1)	0.561
Vertebral artery CeAD^*^	54 (55.7)	29 (70.7)	25 (44.6)	0.009
Internal carotid artery CeAD^*^	43 (44.3)	12 (29.3)	31 (55.4)	0.009
Multi-segments occlusion^*^	33 (34.0)	17 (30.4)	16 (39.0)	0.250
Minor trauma^*^	28 (28.9)	13 (31.7)	15 (26.8)	0.393
Recent infection^*^	13 (13.4)	3 (7.3)	10 (17.9)	0.100
***Treatment***				
Hyperacute treatment				0.763
Heparin^*^	91 (94.8)	38 (92.7)	53 (94.6)	
Antiplatelets^*^	6 (6.2)	3 (7.3)	3 (5.4)	
Medication at discharge				
Antiplatelets^*^	15 (15.5)	7 (17.1)	8 (14.3)	0.460
Vitamin K Antagonists^*^	72 (74.2)	28 (68.3)	44 (78.6)	0.182
DOAC^*^	1 (1.0)	0 (0.0)	1 (1.8)	0.577
Antihypertensives^*^	27 (27.8)	15 (36.6)	12 (21.4)	0.079
Statin^*^	25 (25.8)	13 (31.7)	12 (21.4)	0.182
Days of hospital stay^†^	11.0 (8.0, 16.0)	10.0 (7.0, 15.8)	11.0 (9.0, 17.0)	0.244

^*^ values for *N* (%)

^†^ values for median (1st, 3rd quartile)

*P* value for difference between those with and without recanalization

*CeAD* cervical artery dissection; *mRS* modified Rankin Scale; *NIHSS* National Institutes of Health Stroke Scale; *DOAC* direct oral anticoagulants

Those with recanalization more commonly were female and had internal carotid artery CeAD, which corresponded to both characteristics being independently associated with recanalization in logistic regression analysis (female OR 0.41 [0.18, 0.97]; *P* = 0.043/internal carotid artery dissection OR 0.33 [0.14, 0.78]; *P* = 0.012). The groups did not differ in prior medical treatment, type of antithrombotics in the hyperacute phase or at discharge as well as stroke severity scales or evidence of local symptoms. However, those with spontaneous recanalization less frequently presented with cerebral ischemia at admission. The median time to recanalization in our entire cohort was 90.0 (1st, 3rd quartile: 15.0, 154.5) days, with 14/56 (25.0%) showing recanalization prior to hospital discharge (i.e. early recanalization) and 42/56 (75.0%) after discharge (i.e. late recanalization). Of note, 4/56 (7.1%) recanalizations occurred more than 1-year after initial occlusion.

Table 2 presents follow-up data as well as rates of outcomes of interest in individuals with and without recanalization of initial CeAD-related occlusion.

**Table 2:** Outcome characteristics of the entire cohort as well as differences between those with and without spontaneous recanalization of initially occlusive CeAD

	Entire cohort	No recanalization	Recanalization	*P* value
*N*	97	41	56	
***Follow-up***				
Years of follow-up^‡^^(years)^	8.2 (1.58, 12.8)	7.7 (0.3, 12.5)	8.2 (3.1, 14.0)	0.224
Number of vessel recanalization follow-ups^‡^	11.0 (8.0, 19.0)	10.0 (7.0, 17.8)	12.0 (8.5, 19.0)	0.452
Number of clinical outcome follow-ups^‡^	3.0 (2.0, 4.0)	3.0 (2.0, 4.0)	3.0 (2.0, 4.8)	0.720
mRS at last clinical follow-up^‡^	0.0 (0.0, 1.0)	0.0 (0.0, 1.0)	0.0 (0.0, 1.0)	0.147
***Outcomes***				
Cerebral ischemia†	18 (18.6)	11 (26.8)	7 (12.5)	0.064
Symptomatic ischemia†	10 (55.6)	3 (27.3)	7 (100.0)	0.317

^†^Values given as N (%)

^‡^Values given as median (1st, 3rd quartile)

*P* value for difference between those with and without recanalization

**Fig. 1:**
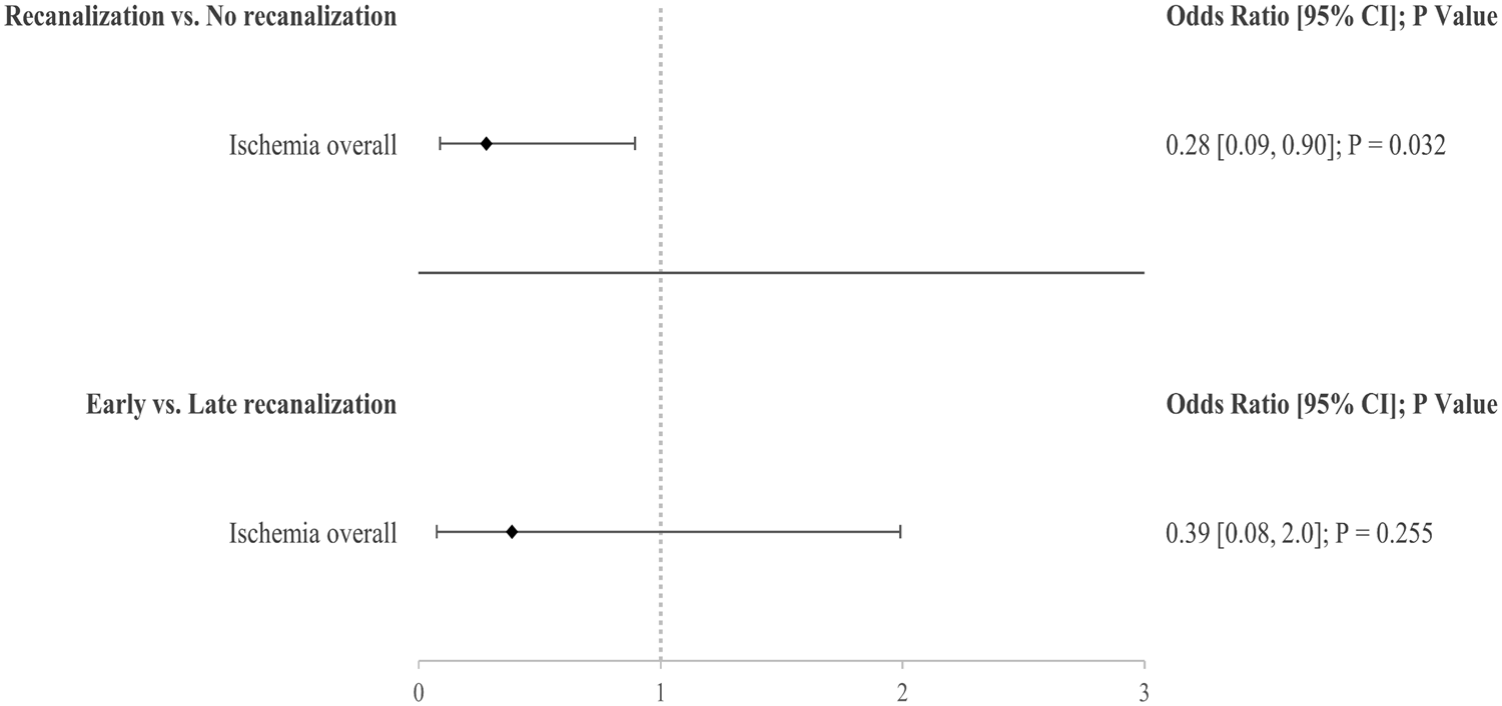
Association of spontaneous recanalization overall as well as its time point on occurrence of cerebral ischemia (including symptomatic or clinically silent) upon follow-up

Overall, when compared with individuals with persistent occlusions, those with spontaneous recanalization did not differ in years of follow-up as well as per-patient number of cerebrovascular- (i.e. recanalization focused) or clinical (i.e. outcome focused) follow-ups. 18/97 (18.6%) patients in our entire cohort with occlusive CeAD had ischemic stroke upon follow-up. 14/18 (77.8%) of these ischemic events were diagnosed within the first 30 days after CeAD diagnosis while 4 cases were diagnosed at 58, 192, 284, 714 days. Those with and without recanalization did not differ in rates of symptomatic strokes, which relates to there being no difference in follow-up mRS as well. In unadjusted group comparison, a trend towards those with spontaneous recanalization less frequently having any stroke upon follow-up, which included silent as well as symptomatic ischemia (26.8 vs. 12.5%, *P* = 0.064) was found. Upon adjusting for biological sex and CeAD-related vessel occlusion localization (i.e. internal carotid or vertebral artery), this became significant in logistic regression analysis (Fig. 1). Patients with early or late recanalization did not differ in follow-up characteristics (Table S-3) and time point did not have a significant impact on occurrence of unfavorable outcomes in our cohort (Fig. 1). The results of both overall and time-point specific recanalization did not change when additionally adjusting for hyperacute treatment (data not shown).

## Discussion

The key findings of this observational cohort study are that (1) about one-third (35.4%) of CeAD patients have initially occlusive vessel pathologies (Table S-1). (2) In more than half (56/97, 57.7%) of patients with occlusive CeAD not undergoing acute revascularization measures spontaneous recanalization occurs over time (Table 1). (3) Internal carotid artery dissection and female sex are the only patient characteristics independently associated with recanalization. Most importantly however, (4) spontaneous recanalization was associated with individuals less frequently suffering stroke upon follow-up (Fig. 1).

Previous studies have shown that occlusive CeAD is associated with higher risk of cerebral ischemia at admission, increased risk of stroke recurrence and subsequently with worse functional outcome compared to nonocclusive CeAD [14, 15, 20]. This is troublesome as patients with occlusive CeAD are routinely managed conservatively due to acute revascularization strategies still being a matter of debate [2, 6, 16, 21]. For instance, the net clinical benefit of EVT is unclear in CeAD and intravenous thrombolysis seems safe and may be beneficial in those treated within the label [5, 22–24]. In terms of EVT, the clinical concerns are especially relevant in isolated cervical artery occlusion without intracranial large vessel occlusion or in initially oligosymptomatic patients. Further, the effect of acute stenting in cases of CeAD related tandem occlusions has been explored in a recent analysis of the multicenter retrospective STOP-CAD study, which could not definitively give a recommendation on opportune management and therefore called for future trials [6]. Yet, in case of isolated CeAD-related cervical artery occlusion, the potential complication of endovascular procedures has to be weighed against the likelihood of spontaneous recanalization [25, 26]. Within our observational cohort, we emphasize on issues raised by literature. For one, patients with initial occlusion compared with those without had higher rates of cerebral ischemia at admission (88.8% vs. 60.8%, Table S-1) and a high overall risk of stroke upon follow-up (18.6%). The low number of individuals in total undergoing acute revascularization measures within our cohort of occlusive CeAD patients (19/116, 16.4%) further illustrates the paucity of clear recommendations for clinicians in the hyperacute treatment setting of CeAD (Table S-2) [21]. Within our study cohort, revascularization measures seem to be reserved for those with severe ischemic strokes of the anterior circulation.

Concerning our main study cohort, more than half of conservatively treated patients with initial occlusive CeAD showed spontaneous recanalization over time with the majority (42/56, 75%) occurring after hospital discharge (in some cases [4/56, 7.1%] even later than 12 months after the index event). Solely internal carotid artery CeAD and female sex were independent factors for the occurrence of spontaneous recanalization (Table 1). Recanalization overall was associated with a lower occurrence rate of stroke upon follow-up (Fig. 1) with ischemic events being diagnosed within 30 days after CeAD diagnosis in most of our patients (77.8%). The likelihood of suffering stroke seemed to be lower in those with earlier recanalization, however, the sample size of our study was considerably underpowered to emphasize on this point (OR 0.39 [CI 0.08, 2.0], Fig. 1). These findings imply that recanalization of initially occlusive CeAD relates to a reduced risk of stroke upon follow-up, even more so if it occurs early. In turn, we additionally emphasize on the fact that patients with persistent occlusion seem to be an at risk population of CeAD in need for optimal management, thus calling for future trials addressing optimal medical treatment in this setting [8, 14]. As trials investigating optimal care of CeAD are inconclusive to date, our findings may assist in the design of future trials [2].

Strengths of our study are the complete long-term intra-individual clinical as well as imaging follow-up. In applying our data, we are able to differentiate and elaborate on unfavorable clinical as well as radiological outcomes and put our findings into context for long-term care of CeAD patients. ProSect study represents one of the largest single-center observational cohorts of CeAD and encapsulates high level of completeness of data as well as follow-up we hope to mediate this concern. We acknowledge that our study has limitations. First, the findings of our study are exploratory and therefore should be interpreted with caution. This is especially true concerning the missing effect of medical treatment on the occurrence of spontaneous recanalization rate and unfavorable clinical outcomes. Our data derives from a prospective single-center cohort that implemented a clinical care pathway for CeAD treatment. Therefore, antithrombotic treatment did not differ between any of the investigated subgroups. In addition, the time-point at which spontaneous recanalization as well as unfavorable clinical outcomes were identified may also be skewed due to study specific imaging assessments at 3- and 12-months post-CeAD diagnosis. Further, findings of subgroups, especially those considering time-point of spontaneous recanalization (i.e. early vs. late recanalization) are hampered by limited sample size.

In conclusion, we provide evidence that spontaneous recanalization in initially occlusive CeAD is frequent and associated with lower rates of stroke upon follow-up. These results indicate that persistent occlusion in patients presenting with occlusive CeAD is a risk factor for occurrence of cerebral ischemia in the long term; thus, calling for future trials addressing optimal medical treatment in this setting.

## Supplementary Information

Below is the link to the electronic supplementary material.

Supplementary file1 (DOCX 33 KB)

## References

[1] Debette S, Leys D (2009) Cervical-artery dissections: predisposing factors, diagnosis, and outcome. Lancet Neurol 8:668–678. 10.1016/S1474-4422(09)70084-5 19539238 10.1016/S1474-4422(09)70084-5

[2] Debette S, Mazighi M, Bijlenga P et al (2021) ESO guideline for the management of extracranial and intracranial artery dissection. Eur Stroke J 6:XXXIX–LXXXVIII. 10.1177/23969873211046475 34746432 10.1177/23969873211046475 PMC8564160

[3] Mayer-Suess L, Peball T, Komarek S et al (2022) Disparities between guideline statements on acute and post-acute management of cervical artery dissection. Rev Cardiovasc Med 23:9. 10.31083/j.rcm2301009 35092201 10.31083/j.rcm2301009

[4] Romoli M, Mosconi MG, Pierini P et al (2021) Reperfusion strategies in stroke due to isolated cervical internal carotid artery occlusion: systematic review and treatment comparison. Neurol Sci 42:2301–2308. 10.1007/s10072-020-04735-5 33037515 10.1007/s10072-020-04735-5 PMC8159826

[5] Traenka C, Lorscheider J, Hametner C et al (2023) Recanalization therapies for large vessel occlusion due to cervical artery dissection: a cohort study of the EVA-TRISP collaboration. J Stroke 25:272–281. 10.5853/jos.2022.03370 37282374 10.5853/jos.2022.03370 PMC10250869

[6] Sousa JA, Rodrigo-Gisbert M, Shu L et al (2025) Emergent carotid stenting during thrombectomy in tandem occlusions secondary to dissection: a STOP-CAD secondary study. Stroke. 10.1161/STROKEAHA.124.048295 39882629 10.1161/STROKEAHA.124.048295

[7] Engelter ST, Traenka C, Gensicke H et al (2021) Aspirin versus anticoagulation in cervical artery dissection (TREAT-CAD): an open-label, randomised, non-inferiority trial. Lancet Neurol 20:341–350. 10.1016/S1474-4422(21)00044-2 33765420 10.1016/S1474-4422(21)00044-2

[8] Kaufmann JE, Gensicke H, Schaedelin S et al (2024) Toward individual treatment in cervical artery dissection: subgroup analysis of the TREAT-CAD randomized trial. Ann Neurol 95:886–897. 10.1002/ana.26886 38362818 10.1002/ana.26886

[9] Kaufmann JE, Harshfield EL, Gensicke H et al (2024) Antithrombotic treatment for cervical artery dissection: a systematic review and individual patient data meta-analysis. JAMA Neurol 81:630–637. 10.1001/jamaneurol.2024.1141 38739383 10.1001/jamaneurol.2024.1141 PMC11091821

[10] Yaghi S, Shu L, Mandel D et al (2024) Antithrombotic treatment for stroke prevention in Cervical Artery Dissection: the STOP-CAD study. Stroke 55:908–918. 10.1161/STROKEAHA.123.045731 38335240 10.1161/STROKEAHA.123.045731

[11] Arauz A, Hoyos L, Espinoza C et al (2006) Dissection of cervical arteries: long-term follow-up study of 130 consecutive cases. Cerebrovasc Dis 22:150–154. 10.1159/000093244 16691024 10.1159/000093244

[12] Charbonneau F, Gauvrit JY, Touze E et al (2005) Diagnosis and follow-up of cervical arterial dissections–results of the SFNV-SFNR study. J Neuroradiol 32:255–257. 10.1016/s0150-9861(05)83147-5 16237364 10.1016/s0150-9861(05)83147-5

[13] Pozzati E, Giuliani G, Acciarri N, Nuzzo G (1990) Long-term follow-up of occlusive cervical carotid dissection. Stroke 21:528–531. 10.1161/01.str.21.4.528 2183403 10.1161/01.str.21.4.528

[14] Mandel DM, Shu L, Chang C et al (2025) Factors associated with stroke recurrence after initial diagnosis of cervical artery dissection. Stroke. 10.1161/STROKEAHA.124.048215 40143807 10.1161/STROKEAHA.124.048215

[15] Traenka C, Grond-Ginsbach C, Goeggel Simonetti B et al (2020) Artery occlusion independently predicts unfavorable outcome in cervical artery dissection. Neurology 94:e170–e180. 10.1212/WNL.0000000000008654 31757869 10.1212/WNL.0000000000008654 PMC6988986

[16] Mayer L, Grams A, Freyschlag CF et al (2020) Management and prognosis of acute extracranial internal carotid artery occlusion. Ann Transl Med 8:1268. 10.21037/atm-20-3169 33178800 10.21037/atm-20-3169 PMC7607089

[17] Mayer-Suess L, Pechlaner R, Barallobre-Barreiro J et al (2020) Extracellular matrix protein signature of recurrent spontaneous cervical artery dissection. Neurology 95:e2047–e2055. 10.1212/WNL.0000000000010710 32887783 10.1212/WNL.0000000000010710

[18] Mayer-Suess L, Frank F, Töll T et al (2022) Head/neck pain characteristics after spontaneous cervical artery dissection in the acute phase and on a long-run. Cephalalgia 42:872–878. 10.1177/03331024221079298 35302384 10.1177/03331024221079298 PMC9315176

[19] von Elm E, Altman DG, Egger M et al (2007) The strengthening the reporting of observational studies in epidemiology (STROBE) statement: guidelines for reporting observational studies. Lancet 370:1453–1457. 10.1016/S0140-6736(07)61602-X 18064739 10.1016/S0140-6736(07)61602-X

[20] Mayer-Suess L, Dejakum B, Ratzinger G et al (2023) Clinical characteristics and outcome in expansive compared with steno-occlusive mural hematoma in spontaneous cervical artery dissection. Int J Stroke 18:1186–1192. 10.1177/17474930231185032 37401395 10.1177/17474930231185032 PMC10676031

[21] Kaufmann JE, Mayer-Suess L, Seiffge D et al (2025) Management of cervical artery dissection: new evidence and future directions. J Neurol 272:426. 10.1007/s00415-025-13166-1 40423824 10.1007/s00415-025-13166-1 PMC12116624

[22] Georgiadis D, Lanczik O, Schwab S et al (2005) IV thrombolysis in patients with acute stroke due to spontaneous carotid dissection. Neurology 64:1612–1614. 10.1212/01.WNL.0000159548.45013.C1 15883325 10.1212/01.WNL.0000159548.45013.C1

[23] Engelter ST, Rutgers MP, Hatz F et al (2009) Intravenous thrombolysis in stroke attributable to cervical artery dissection. Stroke 40:3772–3776. 10.1161/STROKEAHA.109.555953 19834022 10.1161/STROKEAHA.109.555953

[24] Shu L, Akpokiere F, Mandel DM et al (2024) Intravenous thrombolysis in patients with cervical artery dissection. Neurology 103:e209843. 10.1212/WNL.0000000000209843 39298709 10.1212/WNL.0000000000209843 PMC11415265

[25] Baracchini C, Tonello S, Meneghetti G, Ballotta E (2010) Neurosonographic monitoring of 105 spontaneous cervical artery dissections: a prospective study. Neurology 75:1864–1870. 10.1212/WNL.0b013e3181feae5e 20962286 10.1212/WNL.0b013e3181feae5e

[26] Nedeltchev K, Bickel S, Arnold M et al (2009) R2-recanalization of spontaneous carotid artery dissection. Stroke 40:499–504. 10.1161/STROKEAHA.108.519694 19109549 10.1161/STROKEAHA.108.519694

