# Supplementary material for: Impact of spontaneous recanalization of occlusive cervical artery dissection on risk of stroke
Source: J Neurol. 2025 Sep 11;272(9):627. doi: 10.1007/s00415-025-13371-y (PMC12426123; doi:10.1007/s00415-025-13371-y)
Supplement: Supplementary file 1 — Supplementary file1 (DOCX 33 KB) [file 415_2025_13371_MOESM1_ESM.docx]

**Online Supplement**

**Impact of spontaneous recanalization of occlusive cervical artery dissection on risk of stroke**

Lukas Mayer-Suess, MD, PhD*^1^*; Josefin E. Kaufmann, MD, PhD*^2^*; Lukas Scherer, MD*^1, 3^*; Anel Karisik, MD*^1^*; Malik Galijasevic, MD, PhD*^4,5^*; Stephanie Mangesius, MD, PhD*^4,5^*; Elke Ruth Gizewski, MD*^4,5^*; Stefan Kiechl, MD*^1,3^*; Christopher Traenka, MD*^2^*; Stefan T. Engelter, MD*^2^*; Michael Knoflach, MD*^1,3^*

*^1^ Department of Neurology, Medical University Innsbruck*

*^2^ Department of Neurology,* *University Hospital Basel and University of Basel*

*^3^ VASCage, Research Center on Vascular Ageing and Stroke, Innsbruck
^4^ Department of Radiology, Medical University Innsbruck*

*^5^ Neuroimaging Research Core Facility, Medical University of Innsbruck*

**Corresponding author**

Lukas Mayer-Suess, MD, PhD
A-6020 Innsbruck, Anichstraße 35
E-Mail: Lukas.Mayer@i-med.ac.at
Tel: +43/512/504-83127

**Study funding:** Österreichische Nationalbank Anniversary fund (#15644)

Austrian Research Promotion Agency FFG – Project #898252

**Tables:**

| **Table S-1:** Comparison of CeAD patients with and without initial occlusion. | | | |  |
| --- | --- | --- | --- | --- |
|  | **Occlusion** | **No Occlusion** | **P-Value** |  |
| N | 116 | 212 |  |  |
| ***Patient characteristics*** |  |  |  |  |
| Age^†^ | 46.6 (39.5, 52.5) | 42.8 (35.2, 51.2) | 0.020 |  |
| Male^*^ | 70 (60.3) | 120 (56.6) | 0.295 |  |
| mRS prior to CeAD^†^ | 0.0 (0.0, 0.0) | 0.0 (0.0, 0.0) | 0.123 |  |
| ***Medication at admission*** |  |  |  |  |
| Antiplatelets^*^ | 3 (2.6) | 3 (1.4) | 0.388 |  |
| Oral anticoagulants^*^ | 0 (0.0) | 4 (1.9) | 0.158 |  |
| Antihypertensives^*^ | 15 (12.9) | 24 (11.3) | 0.472 |  |
| Statin^*^ | 5 (4.3) | 3 (1.4) | 0.124 |  |
| ***Clinical presentation*** |  |  |  |  |
| Ischemia^*^ | 103 (88.8) | 129 (60.8) | <0.001 |  |
| NIHSS admission^†^ | 1.0 (0.0, 4.0) | 0.0 (0.0, 2.0) | <0.001 |  |
| mRS admission^†^ | 2.0 (1.0, 3.0) | 1.0 (0.0, 2.0) | <0.001 |  |
| Local symptoms^*^ | 89 (76.7) | 166 (78.3) | 0.180 |  |
| Head/neck pain^*^ | 88 (75.9) | 159 (75.0) | 0.378 |  |
| Horner’s^*^ | 15 (12.9) | 39 (18.4) | 0.101 |  |
| Tinnitus^*^ | 2 (1.7) | 12 (5.7) | 0.066 |  |
| Cranial nerve palsy^*^ | 4 (3.4) | 17 (8.0) | 0.068 |  |
| Multiple vessel CeAD^*^ | 16 (13.8) | 36 (17.0) | 0.258 |  |
| Vertebral artery CeAD^*^ | 64 (55.2) | 111 (52.4) | 0.184 |  |
| Internal carotid artery CeAD | 52 (44.8) | 101 (47.6) | 0.184 |  |
| Minor trauma^*^ | 37 (31.9) | 71 (33.5) | 0.427 |  |
| Recent respiratory infection^*^ | 13 (11.2) | 34 (16.0) | 0.146 |  |
| ***Treatment*** |  |  |  |  |
| Acute revascularisation |  |  | 0.023 |  |
| Thrombolysis^*^ | 15 (12.9) | 8 (3.8) |  |  |
| EVT^*^ | 3 (2.6) | 3 (1.4) |  |  |
| Stenting^*^ | 2 (1.7) | 3 (1.4) | 0.603 |  |
| ^*^Values given as N (%)  ^†^Values given as median (1^st^, 3^rd^ quartile)  P-value for difference between those with and without recanalization | | | | |
| CeAD- spontaneous cervical artery dissection; mRS – modified Rankin Scale; NIHSS – National Institutes of Health Stroke Scale; EVT – endovascular thrombectomy | | | | |

| **Table S-2:** Comparison of occlusive CeAD patients treated conservatively or undergoing acute revascularization measures (i.e. iv thrombolysis, EVT, stenting). | | | |  |
| --- | --- | --- | --- | --- |
|  | **Conservative management** | **Acute**  **revascularization** | **P-Value** |  |
| N | 97 | 19 |  |  |
| ***Patient characteristics*** |  |  |  |  |
| Age^†^ | 46.6 (39.8, 52.3) | 46.0 (39.4, 58.6) | 0.547 |  |
| Male^*^ | 57 (58.8) | 13 (68.4) | 0.301 |  |
| mRS prior to CeAD^†^ | 0.0 (0.0, 0.0) | 0.0 (0.0, 0.0) | 0.268 |  |
| ***Medication at admission*** |  |  |  |  |
| Antiplatelets^*^ | 2 (2.1) | 1 (5.3) | 0.434 |  |
| Antihypertensives^*^ | 12 (12.4) | 3 (15.8) | 0.495 |  |
| Statin^*^ | 4 (4.1) | 1 (5.3) | 0.616 |  |
| ***Clinical presentation*** |  |  |  |  |
| Ischemia^*^ | 84 (86.6) | 19 (100.0) | 0.101 |  |
| NIHSS admission^†^ | 1.0 (0.0, 3.0) | 11.0 (2.0, 16.0) | <0.001 |  |
| mRS admission^†^ | 2.0 (1.0, 3.0) | 4.0 (2.0, 5.0) | <0.001 |  |
| Local symptoms^*^ | 78 (80.4) | 11 (57.9) | 0.016 |  |
| Head/neck pain^*^ | 77 (79.4) | 11 (57.9) | 0.022 |  |
| Horner’s^*^ | 14 (14.4) | 1 (5.3) | 0.229 |  |
| Tinnitus^*^ | 1 (1.0) | 1 (5.3) | 0.312 |  |
| Cranial nerve palsy^*^ | 4 (4.1) | 0 (0.0) | 0.470 |  |
| Multiple vessel CeAD^*^ | 15 (15.5) | 1 (5.3) | 0.210 |  |
| Vertebral artery CeAD^*^ | 54 (55.7) | 3 (15.8) | 0.001 |  |
| Internal carotid artery CeAD | 43 (44.3) | 16 (84.2) | 0.001 |  |
| Minor trauma^*^ | 33 (34.0) | 9 (47.4) | 0.112 |  |
| Recent respiratory infection^*^ | 28 (28.9) | 0 (0.0) | 0.081 |  |
| ***Treatment*** |  |  |  |  |
| Acute revascularisation | - | 19 (100.0) |  |  |
| Thrombolysis^*^ | - | 16 (84.2) |  |  |
| EVT^*^ | - | 2 (10.5) |  |  |
| Stenting^*^ | - | 4 (21.1) |  |  |
| ^*^Values given as N (%)  ^†^Values given as median (1^st^, 3^rd^ quartile)  P-value for difference between those with and without recanalization | | | | |
| CeAD- spontaneous cervical artery dissection; mRS – modified Rankin Scale; NIHSS – National Institutes of Health Stroke Scale; EVT – endovascular thrombectomy | | | | |

| **Table S-3:** Outcome characteristics of all patients with recanalization cohort as well as differences between those with early- and late recanalization of CeAD-related occlusion | | | | |
| --- | --- | --- | --- | --- |
|  | **Recanalization** | **Early** | **Late** | **P-Value** |
| N | 56 | 14 (25.0) | 42 (75.0) |  |
| ***Follow-up*** |  |  |  |  |
| Years of follow-up^†^ | 8.2 (3.1, 14.0) | 11.9 (1.0, 16.2) | 7.8 (3.2, 12.6) | 0.374 |
| Number of vessel  recanalization follow-ups^†^ | 12.0 (8.5, 19.0) | 10.5 (6.0, 17.5) | 12.0 (9.0, 19.0) | 0.425 |
| Number of clinical outcome  follow-ups^†^ | 3.0 (2.0, 4.8) | 3.5 (2.0, 5.0) | 3.0 (2.0, 4.3) | 0.584 |
| mRS follow-up^†^ | 0.0 (0.0, 1.0) | 1.0 (0.0, 1.0) | 0.0 (0.0, 1.0) | 0.225 |
| ***Outcomes*** |  |  |  |  |
| Cerebral ischemia^*^ | 7 (12.5) | 2 (14.3) | 5 (11.9) | 0.233 |
| Symptomatic ischemia^*^ | 7 (100.0) | 2 (100.0) | 5 (100.0) | 0.233 |
| *Values given as N (%)  ^†^Values given as median (1^st^, 3^rd^ quartile)  P-value for difference between those with early or late recanalization | | | | |

**STROBE Statement—checklist of items that should be included in reports of observational studies**

|  | Item No. | Recommendation | Page  No. |  |
| --- | --- | --- | --- | --- |
| **Title and abstract** | 1 | (*a*) Indicate the study’s design with a commonly used term in the title or the abstract | 0 |  |
|  |  | (*b*) Provide in the abstract an informative and balanced summary of what was done and what was found | 1 |  |
| Introduction | | | |  |
| Background/rationale | 2 | Explain the scientific background and rationale for the investigation being reported | 3 |  |
| Objectives | 3 | State specific objectives, including any prespecified hypotheses | 3 |  |
| Methods | | | |  |
| Study design | 4 | Present key elements of study design early in the paper | 3-4 |  |
| Setting | 5 | Describe the setting, locations, and relevant dates, including periods of recruitment, exposure, follow-up, and data collection | 3-4 |  |
| Participants | 6 | (*a*) *Cohort study*—Give the eligibility criteria, and the sources and methods of selection of participants. Describe methods of follow-up  *Case-control study*—Give the eligibility criteria, and the sources and methods of case ascertainment and control selection. Give the rationale for the choice of cases and controls  *Cross-sectional study*—Give the eligibility criteria, and the sources and methods of selection of participants | 3-4 |  |
|  |  | (*b*) *Cohort study*—For matched studies, give matching criteria and number of exposed and unexposed  *Case-control study*—For matched studies, give matching criteria and the number of controls per case | 3-4 |  |
| Variables | 7 | Clearly define all outcomes, exposures, predictors, potential confounders, and effect modifiers. Give diagnostic criteria, if applicable | 4-5 |  |
| Data sources/ measurement | 8* | For each variable of interest, give sources of data and details of methods of assessment (measurement). Describe comparability of assessment methods if there is more than one group | 4-5 |  |
| Bias | 9 | Describe any efforts to address potential sources of bias | 4-5 |  |
| Study size | 10 | Explain how the study size was arrived at | 4-5 |  |

Continued on next page

| Quantitative variables | 11 | Explain how quantitative variables were handled in the analyses. If applicable, describe which groupings were chosen and why | 5 |  |
| --- | --- | --- | --- | --- |
| Statistical methods | 12 | (*a*) Describe all statistical methods, including those used to control for confounding | 5 |  |
|  |  | (*b*) Describe any methods used to examine subgroups and interactions | 5 |  |
|  |  | (*c*) Explain how missing data were addressed | / |  |
|  |  | (*d*) *Cohort study*—If applicable, explain how loss to follow-up was addressed  *Case-control study*—If applicable, explain how matching of cases and controls was addressed  *Cross-sectional study*—If applicable, describe analytical methods taking account of sampling strategy | / |  |
|  |  | (*e*) Describe any sensitivity analyses | / |  |
| Results | | | | |
| Participants | 13* | (a) Report numbers of individuals at each stage of study—eg numbers potentially eligible, examined for eligibility, confirmed eligible, included in the study, completing follow-up, and analysed | 6-7, Table 1 |  |
|  |  | (b) Give reasons for non-participation at each stage | / |  |
|  |  | (c) Consider use of a flow diagram | / |  |
| Descriptive data | 14* | (a) Give characteristics of study participants (eg demographic, clinical, social) and information on exposures and potential confounders | 6-7, table 1 |  |
|  |  | (b) Indicate number of participants with missing data for each variable of interest | / |  |
|  |  | (c) *Cohort study*—Summarise follow-up time (eg, average and total amount) | Table 2 |  |
| Outcome data | 15* | *Cohort study*—Report numbers of outcome events or summary measures over time | / |  |
|  |  | *Case-control study—*Report numbers in each exposure category, or summary measures of exposure | Table 2 |  |
|  |  | *Cross-sectional study—*Report numbers of outcome events or summary measures | */* |  |
| Main results | 16 | (*a*) Give unadjusted estimates and, if applicable, confounder-adjusted estimates and their precision (eg, 95% confidence interval). Make clear which confounders were adjusted for and why they were included | 7, Figure 1 |  |
|  |  | (*b*) Report category boundaries when continuous variables were categorized | 6-7 Table 1&2 |  |
|  |  | (*c*) If relevant, consider translating estimates of relative risk into absolute risk for a meaningful time period | / |  |

Continued on next page

| Other analyses | 17 | Report other analyses done—eg analyses of subgroups and interactions, and sensitivity analyses | Supplemental tables |  |
| --- | --- | --- | --- | --- |
| Discussion | | | | |
| Key results | 18 | Summarise key results with reference to study objectives | 8 |  |
| Limitations | 19 | Discuss limitations of the study, taking into account sources of potential bias or imprecision. Discuss both direction and magnitude of any potential bias | 9-10 |  |
| Interpretation | 20 | Give a cautious overall interpretation of results considering objectives, limitations, multiplicity of analyses, results from similar studies, and other relevant evidence | 9 |  |
| Generalisability | 21 | Discuss the generalisability (external validity) of the study results | 9-10 |  |
| Other information | |  | | |
| Funding | 22 | Give the source of funding and the role of the funders for the present study and, if applicable, for the original study on which the present article is based | 11 |  |

*Give information separately for cases and controls in case-control studies and, if applicable, for exposed and unexposed groups in cohort and cross-sectional studies.

**Note:** An Explanation and Elaboration article discusses each checklist item and gives methodological background and published examples of transparent reporting. The STROBE checklist is best used in conjunction with this article (freely available on the Web sites of PLoS Medicine at http://www.plosmedicine.org/, Annals of Internal Medicine at http://www.annals.org/, and Epidemiology at http://www.epidem.com/). Information on the STROBE Initiative is available at www.strobe-statement.org.
